# The Present and Future of Yellow Fever Vaccines

**DOI:** 10.3390/ph14090891

**Published:** 2021-09-01

**Authors:** Clairissa A. Hansen, Alan D. T. Barrett

**Affiliations:** 1 Department of Pathology, University of Texas Medical Branch, Galveston, TX 77555-4036, USA; clahanse@utmb.edu; 2 Sealy Institute for Vaccine Sciences, University of Texas Medical Branch, Galveston, TX 77555-4036, USA

**Keywords:** yellow fever, vaccine, RNA viruses, vaccine platforms, viral re-emergence, chimeric vaccines, vaccine manufacturing, live-attenuated vaccines

## Abstract

The disease yellow fever (YF) is prevented by a live-attenuated vaccine, termed 17D, which has been in use since the 1930s. One dose of the vaccine is thought to give lifelong (35+ years) protective immunity, and neutralizing antibodies are the correlate of protection. Despite being a vaccine-preventable disease, YF remains a major public health burden, causing an estimated 109,000 severe infections and 51,000 deaths annually. There are issues of supply and demand for the vaccine, and outbreaks in 2016 and 2018 resulted in fractional dosing of the vaccine to meet demand. The World Health Organization (WHO) has established the “Eliminate Yellow Fever Epidemics” (EYE) initiative to reduce the burden of YF over the next 10 years. As with most vaccines, the WHO has recommendations to assure the quality, safety, and efficacy of the YF vaccine. These require the use of live 17D vaccine only produced in embryonated chicken eggs, and safety evaluated in non-human primates only. Thus, any second-generation vaccines would require modification of WHO recommendations if they were to be used in endemic countries. There are multiple second-generation YF vaccine candidates in various stages of development that must be shown to be non-inferior to the current 17D vaccine in terms of safety and immunogenicity to progress through clinical trials to potential licensing. The historic 17D vaccine continues to shape the global vaccine landscape in its use in the generation of multiple licensed recombinant chimeric live vaccines and vaccine candidates, in which its structural protein genes are replaced with those of other viruses, such as dengue and Japanese encephalitis. There is no doubt that the YF 17D live-attenuated vaccine will continue to play a role in the development of new vaccines for YF, as well as potentially for many other pathogens.

## 1. Introduction

The disease yellow fever (YF) has been prevented by the use of a live-attenuated vaccine, strain 17D, since 1937. Despite the vaccine being very successful, there are still large outbreaks of YF that put pressure on supply and demand for the vaccine. In this review, we describe the reasons why YF is a re-emerging virus and the current status of the 17D vaccine, and speculate on the future of YF vaccines.

## 2. Yellow Fever Is a Re-Emerging Disease

Yellow fever (YF) is a disease that causes significant morbidity and mortality, but is fortunately vaccine-preventable. Yellow fever virus (YFV), the causative agent of the disease YF, is the prototypical member of the genus *Flavivirus.* The virus is transmitted by mosquitoes and involves primates as amplifying vertebrate hosts. The typical vertebrate hosts are non-human primates (NHPs), but when humans come into contact with the mosquito vectors, they too can act as amplifying hosts. After a human has been bitten by an infected mosquito, the disease typically has an incubation period of 3–6 days. In the first stage of disease, the person will have typical flulike symptoms such as fever, muscle ache, headache, joint pain, and nausea. Next is the period of remission, which lasts for approximately 48 h, and symptoms wane. Approximately one out of every seven infected people will then enter the period of intoxication in which the classic signs of YF present themselves as the virus replicates primarily in the liver (viscerotropism). These symptoms include jaundice, hemorrhages, high fever, dark urine, shock, and organ failure. There are no specific antiviral treatments, and thus treatment relies primarily on palliative care. As the name implies, people who are infected with the virus turn yellow due to liver dysfunction. It should be noted that while the tissue tropism of wild-type YFV is viscerotropism, if YF live-attenuated vaccines revert to virulence, it is usually seen as neurotropic disease, rather than viscerotropic disease.

### 2.1. Epidemiology and Re-Emergence

YF is endemic in 44 countries in tropical South America and sub-Saharan Africa. The virus is maintained in sylvatic and urban transmission cycles. The sylvatic cycle involves *Aedes africanus* (Africa)*, Haemagogus* spp., and *Sabethes* spp. (South America) mosquitoes and NHPs in jungle habitats [1,2]. Risk of human infection and spillover of the virus into the urban cycle increases as humans get closer in proximity to the forests where the sylvatic cycle is present. *Aedes aegypti* mosquitoes are responsible for most of the urban spread of YFV [3,4]. The R_0_ of YF can be as high as 5–7 during urban outbreaks [5]. As climate change worsens and the *Ae. aegypti* habitats expand, regions of the world with people who are naïve to YFV infection are in danger of outbreaks. The danger of urban spread can be put into context with the 2015–2016 outbreak in Angola, where there were 4347 suspected cases, 884 confirmed cases, and 377 deaths [6]. During the outbreak in Angola, cases of YF were imported into China, with a total of 11 confirmed cases and one death. While these were the first cases of YF in Asia, it is important to note that all of the cases were imported from Angola. The high R_0_ and risk of spread to naïve populations highlight the need to control urban outbreaks. Some authors have used the cases in China to suggest the need of vaccination in Asia; however, it is significant that none of the cases in China led to secondary cases, and emphasis should be placed on improved vaccination rates in current endemic countries to prevent spread to new areas.

The case fatality rate of YF varies between 5 and 50% depending on the outbreak [4]. In 2018, there were an estimated 109,000 severe infections and 51,000 deaths due to YF [7]. Recent large outbreaks in Brazil (2016–2018), the Democratic Republic of the Congo (DRC) (2016), Angola (2016), Uganda (2016) [8], and Nigeria (2019) [9] demonstrated why YFV is considered a re-emerging pathogen. In Brazil alone, there were more than 2000 confirmed cases, more than 500 deaths, and more than 4000 epizootics (disease in NHPs) between December 2016 and March 2018 [10]. This epidemic began in the north of the country and moved to southern coastal areas where the virus had not been detected previously, which was further evidence of its re-emergence. There is a need to have a sufficient vaccine supply to keep YF outbreaks under control, and the World Health Organization (WHO) maintains a reserve of approximately 6 million doses for outbreak control. However, it is significant that the Angola outbreak in 2016 resulted in the world’s entire supply of 17D vaccine being exhausted twice, with the same happening again in Brazil in 2018. Both outbreaks were controlled by a dose-sparing strategy in which one-fifth of a dose of vaccine was used for vaccination programs (see below for details). Thus, there are issues of supply and demand for the current live-attenuated 17D vaccine. These issues require either increased production of 17D vaccine or consideration of alternative approaches to new vaccines and vaccination strategies.

### 2.2. WHO EYE Initiative

Following the issues of supply and demand in 2016 and dose sparing, the WHO’s “Eliminate Yellow Fever Epidemics” (EYE) initiative was launched, which lays out a vaccination plan for the next decade that includes routine immunization (RI) and catch-up immunization schedules, as well as preventative mass vaccination campaigns (PVMCs) [11]. The WHO aims to increase 17D vaccine manufacturing to distribute 1.3 billion vaccine doses to endemic countries by 2026, which is a large goal considering that there have only been approximately 1 billion doses distributed in the last 80 years.

The EYE initiative has diligently planned vaccination efforts, but these plans are complicated by the fact that YF epidemics can be difficult to predict and track due to the presence of the sylvatic cycle. The sporadic human cases that have the potential to cause outbreaks typically occur in forested areas, which makes it difficult to obtain samples in real time for either serologic or viral RNA diagnostics. This is important, as the clinical symptoms of YF are similar to those of other viral diseases, including other viral hemorrhagic fevers. Mapping viral incidence and transmission dynamics with geographical modeling and phylogenetics can help with control strategies by providing a clearer view of how YFV spreads between outbreaks.

## 3. Virology

**Figure 1:**
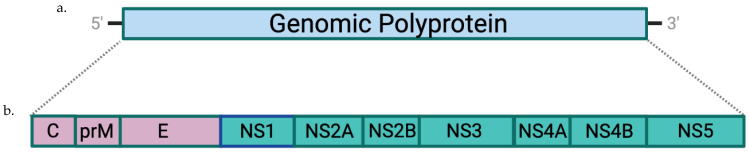
The YFV genome is a positive-stranded RNA (**a**) that is translated into a polyprotein (**b**) that is co- and post-translationally processed into three structural proteins (purple) and seven non-structural proteins (teal).

Members of the Flavivirus genus have a single-stranded, positive-sense RNA genome, which for YFV is approximately 11 kb in length. The genome is translated into a single polyprotein that is co- and post-translationally cleaved by viral and host proteases to generate three structural proteins and seven non-structural proteins (NS1–NS5) (Figure 1). The capsid (C) structural protein forms a nucleocapsid with the viral genomic RNA. The nucleocapsid is surrounded by a host-derived lipid envelope that contains two structural proteins: envelope (E) and membrane (M). The virion is icosahedral and approximately 50 nm in diameter. Virions bud on the endoplasmic reticulum, and the membrane (M) protein is a small 8 kDa protein that facilitates viral maturation and is involved in apoptosis. The E protein is the major immunogen of the virus, facilitates virus-cell fusion, and comprises most of the surface of the virion. It is glycosylated for some flaviviruses and has a molecular weight of approximately 53–56 kDa. The E protein has an ectodomain of approximately 400 amino acids situated on the N-terminus of the approximately 500-amino-acid protein. The ectodomain consists of three domains: EDI, EDII, and EDIII. The YFV E protein has been studied in detail by multiple groups, including studies using human and mouse monoclonal antibodies (mAbs) (reviewed in Davis and Barrett, 2020) [12]. As with other flaviviruses, most neutralizing epitopes are conformational and are found on EDII and EDIII. Interestingly, the 17D vaccine induces few flavivirus cross-reactive neutralizing antibodies, and most neutralizing antibodies are YFV-specific [12].

The non-structural proteins play roles in viral replication and assembly, host innate immune response antagonism, protein cleavage, and more. Their distinct properties are discussed more in-depth in previous articles [13,14,15]. YFV is thought to attach to cells via glycosaminoglycans, but the specific cell receptors that mediate uptake of the virus are unknown [16]. However, wild-type Asibi virus utilizes the classical, clathrin-mediated endocytosis pathway, while live-attenuated 17D vaccine virus has been shown to enter cells in a unique, clathrin-independent mechanism [17]. Following receptor-mediated endocytosis, the virion undergoes pH-mediated fusion with the endosomes, and the genome migrates to the endoplasmic reticulum (ER) [18]. The genome is translated and replicated, and the virions are assembled as they bud through the ER membrane. The virions migrate through the Golgi complex, the M protein undergoes furin-mediated cleavage to transition from its immature form (pre-Membrane (prM)) to the mature form (M) in the virion, and the virions are exocytosed.

## 4. Live-Attenuated 17D Vaccine

Developed in the 1930s by Max Theiler and colleagues, the YF 17D vaccine is one of the oldest live-attenuated vaccines in use today [4]. YFV wild-type strain Asibi was empirically passaged 176 times in embryonic mouse and chicken tissue to yield the live-attenuated 17D vaccine strain [19]. The 17D vaccine is characterized by loss of viscerotropism, loss of neurotropism, and loss of mosquito competence [20].

Unfortunately, the original 17D variant at passage 176 of wild-type Asibi has been lost. Thus, the molecular basis of attenuation has been investigated by comparison of wild-type Asibi virus with current 17D vaccines. There are three substrains of 17D used as vaccines today (17D-204, 17DD, and 17D-213, which are described in detail below) that all share 20 common amino acid substitutions plus four nucleotide changes in the 3’ untranslated region (UTR) when compared to Asibi (Table 1). Briefly, there are nine amino acid substitutions in the structural proteins: one in membrane and eight in the E protein. There are 11 amino acid substitutions in the non-structural proteins, including four in NS2A, two in NS5, and one each in NS1, NS2B, NS3, NS4A and NS4B. There have been a number of studies to investigate the molecular basis of attenuation of the 17D vaccine, and the overall conclusion is that it is multigenic, but the exact amino acids involved in the attenuated phenotype have not been determined. If attenuation proves to be multigenic, this may contribute to the excellent safety record of the 17D vaccine.

Nonetheless, it is also considered likely that the lack of genetic diversity of the 17D virus plays a critical role in the attenuated phenotype [21,22]. RNA viruses are known to have an error-prone replication complex that results in extensive genetic diversity within the RNA population found in virions. This includes wild-type YFV. In contrast, the 17D vaccine has very little genetic diversity, and the overall 17D vaccine from different sources is very homogeneous. The 17D vaccine has been used as a vector to generate other live vaccines in which the prM and E protein genes of 17D virus are replaced with those of other flaviviruses (described below) using a technology known as “ChimeriVax” [23]. These live-attenuated chimeric vaccines would suggest that the 17D non-structural genes are important to the attenuation process.

There have been a number of papers on next-generation sequencing (NGS) of the 17D vaccine that suggest NGS could be used potentially as part of quality control of the vaccine [21,24,25,26,27,28]. However, at the present time, regulators use NGS to look for adventitious agents, and standardizing NGS for quality control of the vaccine would require careful discussion.

The 17D vaccine doses are produced in embryonated chicken eggs using a seed-lot system that was developed in 1945. It is the only vaccine included in the International Health Regulations [29,30]. The primary seed 17D substrain viruses are used to generate secondary seeds that generate vaccine lots to be used for human immunization. These lots are prepared freeze-dried, reconstituted, and 0.5 mL of vaccine is injected either intramuscularly or subcutaneously. Due to the nature of the seed-lot system, there are periodic requirements to make new seed lots. The WHO guidelines state that there must be at least 1000 international units (IU) in each 17D vaccine dose [31], but there is no upper limit. Some vaccine lots by some producers have been shown to have up to 100,000 IU/dose, which is likely due to a combination of improved manufacturing technology over time and the need to ensure a long shelf life (mandated by the WHO to be at least three years) in hot climates [32]. Thus, any new-generation vaccine candidates will require a long shelf life plus higher stability, which would greatly aid vaccination efforts by allowing for lower doses and increased accessibility.

### 4.1. 17D Vaccine Substrains Used Today

Asibi was passaged 204 times in chicken tissue to yield 17D-204, and the vaccine seeds are produced between passages 234–238 [5]. A detailed passage history can be found in previous reviews [4]. On behalf of the WHO, the Robert Koch Institute in Germany took 17D-204 at passage 235 to generate the 17D-213 substrain, which is used at passages 237–238 for vaccine seeds. The 17DD vaccine was generated from the 195th passage of Asibi in chicken tissue and has a distinct passage history from 17D-204, with vaccine seeds used at passages 285–286. There are currently only six vaccine manufacturers worldwide: in the United States of America (17D-204; YF-VAX^®^, Sanofi-Pasteur), Swiftwater, United States, France (17D-204; Stamaril^®^, Sanofi-Pasteur, Marcy l’Etoile, France), Senegal (17D-204; Institut Pasteur, Dakar, Senegal), the People’s Republic of China (17D-204; Tiantan, Wuhan Institute of Biological Products, Wuhan, People’s Republic of China), the Russian Federation (17D-213; Chumakov Institute of Poliomyelitis and Viral Encephalitides, Moscow, Russia), and Brazil (17DD; Bio-Manguinhos/FIOCRUZ, Rio de Janeiro, Brazil). The vaccines manufactured in France, Russia, Senegal, and Brazil are WHO-prequalified to be used in international markets and mass vaccination campaigns. WHO prequalification of vaccines is a comprehensive assessment to ensure the vaccine meets requirements for safety and efficacy in immunization programs used in multiple countries, which is important when the vaccine is distributed to the 44 low- and middle-income countries at risk from YF outbreaks in Africa and South America. The US- and Chinese-manufactured vaccines are only used in domestic markets. It should be emphasized that the two vaccines used in domestic markets are not inferior to those prequalified they just have not applied to the WHO for prequalification to be used internationally. None of the substrains appear to diverge in attenuation phenotype, and multiple non-inferiority trials have shown each substrain to be adequate for protection against YF [4,33,34,35].

### 4.2. The 17D Vaccine: Molecular Mechanisms and Immune Responses

In the 1970s, Mason et al. used NHPs to show the correlate of protection after the YF vaccination was neutralizing antibodies with a titer determined by log_10_ neutralization index (LNI; i.e., constant antibody, varying concentrations of virus) of 0.7 [36]. The plaque reduction neutralization test (PRNT) (i.e., constant virus, varying concentrations of antibody) has mostly replaced the LNI, but the two tests have unfortunately never been validated against each other. This is a major gap in knowledge, but different studies have estimated the LNI of 0.7 is equivalent to a 50% PRNT value of 1 in 20 to 1 in 40 [37]. Nonetheless, many clinical trials use a cut-off for protective immunity based on a neutralization titer of 1 in 10 by a 50% PRNT assay. If and when new YF vaccines are developed, it would be ideal to have a standardized neutralization assay to validate the vaccines from the beginning.

Despite being developed 85 years ago, the vaccine remains highly effective, with studies showing at least 35 years of immunity after only one dose, and with detectable antibodies up to 40 years [38,39,40]. The molecular basis of long-lasting immunity is still poorly understood. Studies have reported that 75–100% of those vaccinated with 17D more than 10 years post-vaccination continue to have protective levels of antibodies against YFV [41]. It is widely accepted that the 17D vaccine confers lifelong immunity in the majority of vaccinees.

It is unknown if lacking neutralizing antibodies necessarily means there is a lack of protective immunity, as other types of immunity may be intact [40]. Some studies have shown that a significant portion (but not all) of those who have received a primary YF vaccination do not form an anamnestic response after a booster dose [42,43,44]. This suggests that the memory immune response against YFV is sterilizing, and/or that the cellular memory response proliferates very quickly to clear the virus [40].

Children often have faster waning antibodies after 17D vaccination than adults [40,45,46]. In one study based in Southeast Brazil, it was found that seropositivity in children aged nine months to 12 years dropped from 86.7% in newly vaccinated individuals to 42.2% in those who had been vaccinated from 73 to 100 months prior to antibody testing [46]. This suggests that overall immunity wanes more quickly in children who are vaccinated in this age range than in adults.

Titers of neutralizing antibodies are often reduced in human immunodeficiency virus-positive (HIV^+^) individuals [47,48]. Data from a Swiss HIV cohort study showed that HIV-infected people immediately after 17D vaccination had lower levels of neutralizing antibodies against YFV when compared to non-HIV-infected people [48]. In addition, many of these individuals had faster waning immunity than non-HIV-infected people in longer-term studies, with seropositivity lowering to 75% at 10 years post-vaccination [49]. In a meta-analysis of studies on 17D vaccination of people living with HIV, it was found that 97.6% of the population seroconverted. Between one and 10 years after vaccination, a mean of 72% of this population still had neutralizing antibodies, and after 10 years, a mean of 62% still had neutralizing antibodies [50]. However, HIV-infected individuals who had suppressed plasma HIV RNA at the time of 17D vaccination had up to 100% seropositivity at 10 years post-vaccination, which is comparable to non-HIV-infected individuals [49]. This suggests that control of HIV infection is very important to maintenance of YF vaccine-induced immunity.

Vaccination of HIV-infected individuals with 17D appears to be safe and mostly effective for those who have high CD4^+^ T-cell counts and do not otherwise have severe immunosuppression [48,49]. There is even some in vitro evidence that infection of macrophages and CD4^+^ T cells with 17D inhibits HIV replication through various mechanisms in innate and adaptive immunity and host gene expression [51].

A small study analyzed the peripheral blood mononuclear cells (PBMCs) and serum of six individuals at various timepoints post-17D vaccination [39]. Samples were also collected from 99 subjects at >10 years post-vaccination. By day 12, YF-tetramer^+^ CD8^+^ T cells were detectable and remained present through day 180. These T cells differentiated to become mostly memory T cells (CD45RA^−/+^CD27^−^) by day 28. Through day 180, CD45RA^+^CD27^−^ and CD45RA^+^CD27^+^ T cells persisted. In terms of a long-lasting response, these CD8^+^ T cells persisted for at least 18 years, while YF-specific antibodies persisted up to 40 years post-vaccination and were not increased by booster doses.

The first “systems vaccinology” studies utilized the 17D vaccine and applied modern immunological techniques to study the immune response to 17D vaccination [52,53]. These studies utilized gene signatures to investigate and predict the immune response following 17D vaccination. Vaccination induced genes that regulated complements, the inflammasome, interferons, and adaptive immunity, including complement protein C1qB and eukaryotic translation initiation factor 2 alpha kinase 4, were found to correlate with YF-17D CD8^+^ T-cell responses, while B-cell growth factor TNFRS17 was found to predict the neutralizing antibody response.

In a study by Wec et al. (2020), the B-cell responses of two human subjects after 17D vaccination were evaluated for a year [54]. They characterized YFV E protein-specific monoclonal antibodies (mAbs), as well as memory B cells (MBCs) and plasmablasts (PBs). As found previously in clinical studies, both subjects had serum-neutralizing activity by day 10 post-vaccination that persisted throughout the study. This supported the International Health Regulations that the YF vaccination certificate is valid 10 days post-vaccination [47], They also found that the PB populations at days 10 and 14 were 10- to 20-fold higher than PB populations pre-vaccination, and that these populations had high rates of somatic hypermutation (SHM) [54], which was found to be important, as earlier iterations of the antibodies produced by these B cells had much lower binding affinities to the YFV E protein. It was also found that the MBC response continues to evolve with SHM and germinal center activity for six to nine months. By day 14, up to one-third of the Abs produced had neutralizing activity. This relatively quick production of high-affinity Abs may in part explain why protection against YF occurs so early post-vaccination.

The early memory B-cell response could be differentiated from the late response, as the early response had class-switched immunoglobulins and classical IgM, while the late memory B-cell response is characterized by class-switched immunoglobulins, atypical IgM, and IgD-producing memory B cells [54]. The early response waned rapidly before day 90, but the late response persisted throughout the year-long study. As expected, the majority of the mAbs produced (neutralizing or otherwise) primarily targeted epitopes that are proximal to the fusion loop in EDII. Some of these mAbs were cross-reactive with other flaviviruses, but there was little to no cross-neutralizing activity, as seen in previous studies.

### 4.3. Current WHO Recommendations

The WHO has published detailed recommendations to assure the quality, safety, and efficacy of the yellow fever 17D vaccine. The most recent update was published in the WHO Technical Report Series in 2013 [31]. These recommendations specify that, currently, the only approved vaccine virus is live-attenuated 17D. Furthermore, the vaccine can only be produced in embryonated chicken eggs, and the only approved animal model for safety testing is the NHP, specifically Rhesus macaques and Cynomolgus macaques. The WHO recommends vaccination for those who live in endemic areas, and lists the 17D vaccine as an essential childhood vaccination for children of at least nine months of age in these areas; this age is based on early safety studies. The 17D vaccine has been approved by the WHO to be co-administered with 10 other vaccines, which helps ease the burden of scheduling childhood vaccinations [4,13,47]. The vaccine is also recommended for lab personnel working with YFV and people traveling to endemic areas. In fact, YF is the only disease that requires an international vaccination certificate under International Health Regulations [30].

The 17D vaccine is contraindicated for those who have hypersensitivity reactions to 17D vaccine components, including eggs [47]. It is also contraindicated for children under nine months of age, lactating individuals with babies under nine months of age, and for those 60 years and older, as these groups are at a much higher risk of developing vaccine-associated neurotropic disease (YEL-AND) and viscerotropic disease (YEL-AVD). It should be emphasized that both are very rare safety issues. Critically, YEL-AND and YEL-AVD have only been seen in primary vaccinees.

Little is known about what causes certain people to develop YEL-AVD. However, research to date suggests that certain host genetic factors may make individuals more susceptible to these outcomes [55,56,57,58] In contrast, there is evidence to suggest that YEL-AND is due to mutation in the 17D virus. Jennings et al. showed that a virus isolated from a case of post-vaccinal encephalitis had mutations in the E protein (E-155 and E-303) [59].

It has often been questioned whether or not there is a need for booster doses of the 17D vaccine, because the duration of protective immunity is not completely clear and is still the subject of debate [40]. At the present time, the WHO has deemed 10-year booster doses of the 17D vaccine unnecessary, and removed the requirement for these boosters in 2013 [40,60]. Nonetheless, due to their compromised immune response, the United States Advisory Committee on Immunization Practices (ACIP) recommends that HIV^+^ individuals be given booster doses of the 17D vaccine every 10 years or as necessary [61]. Others who are more likely to be prescribed precautionary booster doses are those who had their last 17D vaccine more than 10 years prior, who are traveling to endemic areas that are especially higher-risk based on the season, current outbreaks, rural proximity, or if they will be spending a prolonged period of time in that area [61]. While the 17D vaccine is recommended for people over the age of nine months and less than 60 years, young children, particularly those <2 years old, have been shown to have lower seroconversion rates than adults after one dose of the vaccine [45].

### 4.4. Dose Sparing

As stated above, due to the recent outbreaks of YF, world vaccine supplies were exhausted twice in early 2016 [5]. This was, in part, due to the requirement to produce the vaccine in specific pathogen-free embryonated chicken eggs. Thus, the six vaccine manufacturers combined can only produce a maximum of about 80–120 million doses/year, though the real number of vaccines produced is usually 33–80 million doses, and the production capabilities vary greatly from year to year for a variety of reasons. It was clear that there was insufficient vaccine to immunize everyone at risk in Angola and DRC in 2016. Fortunately, the Brazilian manufacturer, who produce the 17DD substrain vaccine, had published papers describing use of fractions of a full dose in clinical trials [62,63]. The neutralizing Ab response to 587 IU was indistinguishable from that obtained with a full dose of 17D vaccine (27,476 IU). This gave confidence that the 17DD substrain vaccine produced in Brazil could be utilized at a fraction of a full dose. Since the vaccine is given in a volume of 0.5 mL, it was decided to use the vaccine at 1/5th of a full dose (i.e., a 100 µL volume; such a volume would contain >1000 IU of vaccine), as this could be administered accurately using a tuberculin syringe.

In August 2016, the DRC and Angola received emergency permission, as an off-label use, from the WHO to use fractional doses of the 17DD vaccine at 1/5 of the standard dose in order to vaccinate as many people as possible with dwindling supplies [42]. The DRC vaccinated 7.5 million people with this method, and a study evaluating immunogenicity in a cohort immunized with the fractional dose found it to be indistinguishable from a full dose of vaccine [42]. When vaccine stockpiles were again exhausted in December 2016, Brazil vaccinated 24 million people with fractional doses in 2017–2018 [6,10].

In a 2017–2018 non-inferiority trial based in Mbarara, Uganda and Kilifi, Kenya, fractional dosing of all four WHO-prequalified 17D vaccines were tested in eligible adults [35]. The primary outcome of the study was the proportion of participants with seroconversion at 28 days post-vaccination. Non-inferiority was defined as a less than 10% decrease in seroconversion with fractional doses. All fractional-dose vaccines met the non-inferiority criteria, indicating all 17D vaccines could be used for dose sparing, if required. These results were promising, and some countries have considered fractional dosing outside of emergency situations, but this would be considered an off-label use of the vaccine at the present time. While these studies indicated that fractional dosing could be a viable path forward with vaccination, they all still only tested doses that contained over 1000 IU. Lower doses need to be studied in order to establish an acceptable lower threshold of IU for 17D vaccines. In fact, a complementary study is being undertaken with the 17D-204 vaccine produced at the Institut Pasteur de Dakar, Senegal, recruiting volunteers from Kenya and Uganda to be vaccinated with either a full dose, 1000 IU, 500 IU, or 250 IU of the vaccine (ClinicalTrials.gov Identifier: NCT04059471) [64]. In addition, there was a non-inferiority trial studying full doses and 1/5 fractional doses of the Chumakov Institute 17D-213 vaccine in HIV-infected adults and children aged nine months to five years (ClinicalTrials.gov Identifier: NCT02991495).

### 4.5. Animal Models

Animal models for YFV have been reviewed [65]. Cynomolgus and Rhesus macaques are the only accepted animal models for evaluation of the safety of the 17D vaccine virus in current WHO recommendations. However, it is important to note that there are few wild-type YFV strains that cause viscerotropic disease in NHPs, and passage in cell culture results in even more loss of viscerotropism in NHPs [66]. Therefore, the NHP model is most often used to assess neurotropic disease following direct inoculation of virus into the brain, while the method of assessing viscerotropic disease in these animals is to measure viremia. NHPs are also used in a neurovirulence assay to evaluate vaccine seeds for safety and immunogenicity. The lack of an approved animal model for viscerotropic disease, other than viremia, poses a problem for research on YF.

There are currently no WHO-accepted mouse models for YF. However, mice prove useful in studying neurotropism and immune responses against YFV. Immunocompetent mice succumb to neurotropic, rather than viscerotropic, disease caused by all strains of YFV, and whether intracerebral or peripheral routes of inoculation are used depends on the age of the mice [67]. In comparison, immunocompromised mice do distinguish neurotropic (live vaccine strains) from viscerotropic (wild-type strains) disease. Various immune system knock-out mice (interferon (IFN)-αβ receptor knock-out, IFN-αβγ receptor knock-out, and signal transducer and activator of transcription 1 (STAT1) knock-out) present with distinct pathologies when infected with 17D or wild-type strains of YFV. For example, when IFN-αβ receptor knock-out (also known as A129 mice on a S129 background and IFNAR^−/−^ mice on a C57BL/6 background) (or STAT1^−/−^ knock-out) mice are infected with the 17D virus by a peripheral route, few if any animals die, whereas wild-type strains cause lethal viscerotropic disease [68]. In comparison, in IFN-αβγ receptor knock-out mice (also known as AG129 mice), the 17D virus causes lethal neurotropic disease, and wild-type strains cause lethal viscerotropic disease when inoculated by a peripheral route [69]. Due to these distinct pathologies, it is possible that certain mice will be useful in vaccine testing and development for new YF vaccines [70].

As with mice, YFV is also neurotropic in hamsters [71]. However, wild-type strains have been adapted to hamster liver and show viscerotropic disease in hamsters. These strains include Jimenez hamster passage 10, which causes 80% mortality and high viremia [72], and Asibi hamster passage 7, which causes 100% mortality and moderate viremia [73]. These hamsters are mostly used in the evaluation of antiviral candidates, but have been used to evaluate vaccine candidates as well [37,74,75]. Even with NHPs, mice, and hamsters, more research is needed for animal model development for studying YF vaccine candidates.

## 5. The Future of YF Vaccines

With a finite vaccine seed-lot system, limited vaccine manufacturing capabilities using embryonated chicken eggs, climate change pushing mosquito habitats to new regions, and recent epidemics exposing issues in rapid vaccine dissemination, the need to develop new YF vaccine candidates grows. A more shelf-stable vaccine could mean that more doses could be generated with fewer IU per dose. YF vaccine candidates in development include inactivated vaccines [74,76,77,78], recombinant vaccinia constructs [75,79,80], plasmid-vectored DNA constructs [81,82,83,84], virus-like particles (VLPs) [85], mRNA vaccines [86], synonymous mutations in live-attenuated vaccines [87], and plant-produced subunit vaccines (Table 2) [88]. The 17D genetic backbone has also been utilized in the development of multiple chimeric vaccines for other pathogens (Table 3).

### 5.1. Other Platforms in Development for Candidate YF Vaccines

#### 5.1.1. Inactivated Vaccines

Over the years, there has been periodic interest in inactivated YF vaccines based on the successes of such vaccines for the related flaviviruses JE and tick-borne encephalitis (TBE), which are manufactured in Vero and chick embryo fibroblasts, respectively [107,108,109,110]. An inactivated vaccine would overcome the problems of YEL-AVD and YEL-AND with the live 17D vaccine, would allow those over 60 to receive a primary dose of vaccine, and would have advantages for the travel vaccine market. However, multiple doses and use of an adjuvant would likely be required to induce a protective immune response, as is necessary for inactivated JE and TBE vaccines. No inactivated YF vaccines have been licensed to date, and those developed have involved manufacture of the 17D virus only, in either chick embryo fibroblasts or monkey kidney Vero cells, and inactivation with formalin or beta-propiolactone (BPL). There is limited information available on the durability of protection of inactivated YF vaccine candidates, but early tests show that seroconversion exceeds typical standards [4].

XRX-001 (an inactivated YF vaccine candidate) was generated by purifying and inactivating YF-VAX that was cultured in Vero cells with BPL and adsorbing it to aluminum hydroxide [74]. Preclinical studies in mice, hamsters, and cynomolgus macaques showed this inactivated vaccine candidate to be highly immunogenic, with antibody titers similar to those induced by live 17D vaccine (YF-VAX). Two doses of XRX-001 induced higher levels of antibodies than YF-VAX. After vaccination with one or two doses, hamsters were challenged with the wild-type Jimenez P10 strain of YFV, and all were protected from morbidity and mortality. A phase I clinical trial investigated both safety and immunogenicity of two doses of XRX-001 [89]. 100Of the subjects, 100% of those who received the higher dose generated neutralizing antibodies against YFV after two doses of the vaccine candidate, compared to only 88% of those who received the lower dose. The antibody titers exceeded the minimum protective level. No significant safety issues were found.

A similar strategy was used to develop an inactivated YF vaccine candidate from the 17DD substrain by Bio-Manguinhos/FIOCRUZ [76,77]. The 17DD vaccine was propagated in bioreactors with serum-free Vero cells and was subsequently purified by ion-exchange chromatography, inactivated with BPL, and adsorbed to aluminum hydroxide [76]. After vaccinating C57BL/6 mice with three doses of the inactivated 17DD vaccine, all of the mice had high neutralizing antibody titers and survived a neurovirulence challenge of an intracerebral inoculation of 17DD virus [77]. This vaccine candidate shows promise in terms of scale-up capabilities and antigen structure preservation. It was estimated that one 320 L bioreactor could generate up to one million doses, while the current egg-based technology would have to use up to 5000 eggs for the same amount of product [76]. However, the number of doses needed for protective immunity will have to be considered. Researchers at the Chumakov Institute has also been working on an inactivated vaccine, and they too have grown their vaccine virus in Vero cells and inactivated the virus using BPL. As with the FIOCRUZ vaccine candidate, immunization of mice (BALB/c) with the inactivated vaccine induced high titers of neutralizing antibodies, which exceeded those induced by live vaccine virus [78].

#### 5.1.2. Vaccinia Constructs

Recombinant vaccinia viruses have been constructed to express proteins of 17D, including structural and non-structural proteins [75,80,90]. The recombinant non-replicating modified vaccinia Ankara (MVA) construct containing 17D prM and E was able to stimulate the production of neutralizing antibodies, and protected mice against lethal challenge with YFV [80]. Protection with a low dose of 105 TCID50 was determined to be equivalent to that of 17D. The recombinant virus had a slightly better mouse safety profile than 17D, since it did not cause mortality when injected intracerebrally. Also, pre-existing immunity to vaccinia virus did not appear to hinder the development of a protective immune response to YFV. Another MVA construct containing 17D prME induced neutralizing antibodies in hamsters and protected them from challenge with wild-type Jimenez strain [75]. This vaccine candidate has progressed to phase I clinical evaluation (NCT02743455), but no results have been published.

#### 5.1.3. Plasmid-Launched Live-Attenuated Vaccines (PLLAV)

A number of groups have developed plasmid-based infectious clone systems to study YFV [111,112]. This plasmid includes the entire YFV cDNA genome downstream from an SP6 bacteriophage promoter. This system has been investigated to generate plasmid-launched live-attenuated vaccines (PLLAV) because a DNA form of a live-attenuated RNA virus vaccine would be much more stable than the live virus version, and would have advantages for chemistry, manufacturing, and controls compared to a traditional live-attenuated vaccine (reviewed by Pushko et al.) [113]. Briefly, the PLLAV consists of a full-length cDNA of the 17D genome together with a promotor to transcribe the cDNA inside a cell. The PLLAV is transfected into cells, or an animal, and the cDNA is transcribed in cells by host enzymes to produce viral RNA genome that is translated in the cells to replicate virus and generate the 17D vaccine virus. In theory, the virus thus produced can amplify and spread like the genuine live vaccine, causing a self-limiting infection and finally inducing immunity in a vaccinated subject.

One PLLAV approach uses the “immunization DNA” (iDNA^®^) platform, which was generated by inserting the CMV major immediate-early promoter upstream of the YF 17D cDNA genome to form the pYF17D-5 iDNA plasmid. This was then manipulated to inactivate cryptic promoters, and the resulting iDNA plasmid was called pYF17D-16 [82]. It is capable of being transcribed into the full-length 17D genome, which can launch the 17D virus in host cells. The vaccine virus successfully replicated in vitro after transfection of this iDNA into Vero cells. Safety of this iDNA-derived virus was confirmed in AG129 mice, and was found to be similar to the 17D vaccine virus pathologically, immunologically, and in replication kinetics. Immunogenicity was tested in BALB/c mice by vaccinating them with the iDNA plasmid itself. It was found that the mice produced YFV-specific neutralizing antibodies that were equivalent to or higher than those produced by 17D-vaccinated mice. Similar PPLAVs have also been reported for 17D [83,84] and SARS-CoV-2 chimeric vaccines [106].

#### 5.1.4. Other Plasmid-Vectored DNA Vaccines

Another DNA-vectored vaccine candidate uses only prME of 17D rather than the whole genome [81]. A study on this candidate generated two similar candidates (one with E fused to lysosomal-associated membrane protein signal (LAMP-1) to better target vaccine products to the major histocompatibility complex II) with prME inserted into the p43.2 vector. T-cell and neutralizing-antibody responses to these constructs in C57Bl/6 and BALB/c mice were similar in magnitude and epitope scope to 17DD, with the modified LAMP-1 construct performing best. Vaccinated mice also had a 100% survival rate after intracerebral challenge with YFV.

#### 5.1.5. Virus-Like Particles (VLPs)

VLPs are particles that display viral antigens and lack genetic material, making them replication-incompetent. A multivalent VLP vaccine candidate has been developed for YF, JEV, ZIKV, and Chikungunya (CHIKV) that expresses CprME of the flaviviruses and C-E3-E2-E1 of CHIKV [85]. The viral proteins are expressed on a lentiviral vector that has been stably expressed in 293 T cells under antibiotic selective pressure. Since the C protein of flaviviruses needs to be cleaved from prME by the viral protein NS2B, NS2B must also be expressed in this cell line. It was found that ZIKV NS2B3 effectively cleaved C from prME in ZIKV, YFV, and JEV in this system. To aid vaccine manufacturing scale-up, the cells that stably expressed the VLPs were adapted to grow in suspension. This vaccine candidate was tested in BALB/c mice in monovalent, bivalent, and tetravalent formulations. The monovalent immunizations induced the strongest immune responses to the individual viruses, likely due to the increased amount of antigen for each virus. The tetravalent VLP candidate also stimulated strong neutralizing antibody titers against all four viruses in mice. However, challenge experiments are required to assess the efficacy of the potential vaccine in the mouse model. Manufacturing VLPs at scale has recently been reported for such a candidate vaccine [114].

#### 5.1.6. mRNA Vaccines

The use of mRNA vaccines as a platform technology has been discussed for a number of years. Recently, there have been many developments in stabilizing mRNA molecules for use in vaccines, and multiple COVID-19 vaccines utilize mRNA technology [115,116]. These mRNA vaccines also have the advantage of being relatively quick to develop, since the majority of what is needed for development is the mRNA sequence of the antigen of interest. This sequence can be adjusted relatively quickly if vaccines are to be made against different strains or variants of viruses that already have an mRNA vaccine or for emerging pathogens [86]. The Coalition for Epidemic Preparedness Innovations (CEPI) has recently sponsored CureVac to develop mRNA vaccines for Lassa fever, rabies, and YF using mRNA “printing” technology [117]. In a separate study on mRNA technology, the prME mRNA of YFV was labeled with an orthogonal dual PET–near-infrared (IR) probe to test the trafficking of a YF mRNA vaccine candidate to the draining lymph nodes [86]. Cynomolgus macaques were vaccinated with the YF mRNA vaccine and analyzed with positron emission tomography. It was determined that the vaccine particles migrated to the draining lymph nodes, and that vaccine products were primarily expressed by professional antigen-presenting cells. A YF mRNA vaccine has potential, but more pre-clinical studies are needed before a candidate can move into clinical evaluation.

#### 5.1.7. Codon-Deoptimized Live-Attenuated Vaccines

Codon deoptimization is a method in which synonymous mutations are made to codons to render them suboptimal in certain hosts. It does not change the amino acid sequence of the proteins, but rather makes it more difficult for the host cell to generate those proteins due to the suboptimal codons. Deoptimizing multiple codons can attenuate viruses, as well as lower the risk of reversion and recombination of the attenuated virus [118,119]. Through a re-encoding strategy that aimed to have a low impact on CpG/UpA composition and secondary RNA structures, multiple mutants of the Asibi and Ap7M (hamster-adapted) strains of YFV were generated to have 100–400 synonymous transition mutations in the NS2A-to-NS4B coding region of the YFV genome [87]. The in vitro multiplication kinetics of these mutants did not differ from their parent strains, while in vivo, some of the mutants had decreased replicative fitness, and all showed decreased virulence in the hamster model. Interestingly, the mutants with the highest level of attenuation induced protective immunity in the hamster model.

#### 5.1.8. Plant-Produced Subunit Vaccines

In the search for vaccine manufacturing technologies that are simple to scale up, many researchers have turned to plants to produce antigens for vaccines. Plants can post-translationally modify proteins much like other eukaryotes, and there are many different ways to introduce genes into plants for transient expression [120]. The YF E protein gene has been successfully transiently expressed in *Nicotiana benthamiana* [88]. In this study, the YF E gene was either used as a standalone subunit or was genetically fused to engineered lichenase from *Clostridium thermocellum* (LicKM) and was cloned into the pGR-D4 vector. These plasmids were electroporated into *Agrobacterium tumefacians*, which were then introduced to *N. benthamiana* by vacuum infiltration. The E proteins were purified from the plants and used for pre-clinical studies. Both E and E-LicKM induced production of neutralizing antibodies in mice, and 70% of the mice were protected against lethal infection with YFV post-vaccination. Neutralizing antibodies were also produced following immunization of Rhesus macaques. In a post-vaccination challenge of Rhesus macaques with 17DD, the neutralizing antibody titers increased. Because the protection from this plant-based subunit vaccine was inferior to vaccination with approved 17D substrains, it is likely that this vaccine candidate will require further development in order for it to progress as a vaccine candidate.

### 5.2. New Manufacturing Protocols

The current 17D vaccines are generated using an embryonated chicken-egg-based technology. Although the technology has improved over time with manufacturing technology and increased virus yields, the limitation of specific pathogen-free eggs is still a bottleneck in manufacturing. There have been studies to adapt the 17D vaccine virus to WHO-approved cell substrates, with most studies focusing on Vero cells [70,74,76,77,89]. In one such study, researchers at Sanofi Pasteur purified RNA from the Stamaril^®^ and YF-VAX^®^ YF vaccines, transfected the genomic RNA into Vero cells in serum-free media, plaque-purified the viruses, and rescued 24 clones/“substrains” of the 17D-204 vaccine [70]. The pre-clinical profile of these substrains was tested in A129 mice (for neurotropism and viscerotropism), OF1 mice (for neurovirulence), and hamsters (for immunogenicity). The substrain with the safest preclinical profile that closely matched that of Stamaril^®^ and YF-VAX^®^ (low neurovirulence, neurotropism, viscerotropism, and high immunogenicity) was chosen for further testing. Working seed lots, master seed lots, and bulk stage lots of this substrain, vYF-247, were generated, and all were tested for immunogenicity and protective efficacy in hamsters. All lots elicited high neutralizing antibody responses. When the hamsters were challenged with a lethal dose of wild-type Jimenez P10 YFV, almost all the non-vaccinated controls succumbed to disease, while the hamsters vaccinated with either YF-VAX or vYF-247 had no morbidity or mortality, and likely had sterilizing immunity against YFV. vYF-247 differs from YF-VAX by two amino acids: E-V480L and NS2A-M65V, which do not match any of the known 17D/Asibi mutations. This candidate has advanced to clinical evaluation for safety and immunogenicity in phase I/II (NCT04142086), and non-inferiority compared to 17D YF-VAX in phase II clinical trials (NCT04942210).

In the current embryonated chicken egg manufacturing protocol, 17D is primarily propagated in the skeletal muscle tissue [121]. To better understand this process and to investigate development of an in vitro vaccine manufacturing protocol, primary cultures of chicken embryo skeletal muscle cells were infected with substrain 17DD. It was found that the in vitro method produced the same infection pattern as the accepted in vivo protocol. 17DD has also been propagated in chicken embryo fibroblast (CEF) cell cultures [92]. In CEF cultures, 17DD grew to at least the minimal titers required for 17D vaccines and was as thermostable as the accepted vaccines. Unlike when 17D is grown in other cell cultures, 17DD grown in CEF did not produce any significant genetic variants. The only borderline significant difference between the CEF 17DD and the seed lot virus was that the CEF viruses had slightly higher clinical scores in the monkey neurovirulence test. These studies further evidence that there are many in vitro manufacturing protocols that should be explored for the production of 17D vaccines.

### 5.3. Rationally-Designed Chimeric Vaccines

There has been a lot of interest in using the live-attenuated 17D vaccine genome as a genetic backbone for vaccines against other viruses. Many of these viruses are flaviviruses including West Nile virus (WNV), Japanese Encephalitis virus (JEV), tick-borne encephalitis virus, Zika virus (ZIKV), and dengue virus (DENV).

Since 2012, two vaccines using the 17D-204 backbone (ChimeriVax technology) have been approved for use: Imojev™ and Dengvaxia^®^, to prevent Japanese encephalitis (JE) and dengue, respectively.

Imojev™ (or JE-CV) is a live-attenuated vaccine for JE and contains prM and E protein genes of JE virus live-attenuated vaccine strain SA14-14-2 in the 17D-204 backbone [93,122,123]. This vaccine represented a number of “firsts”: the first live-attenuated vaccine produced in monkey kidney Vero cells, the first chimeric live-attenuated vaccine, and the first live-attenuated vaccine produced using recombinant DNA technology. The latter required a significant environmental impact statement. Imojev, which is given as a one-dose regimen, was first licensed in 2012, and is currently licensed in 14 countries [94]. Imojev was found to be highly efficacious, and one dose induced protective immunity similar to that of a two-dose inactivated vaccine regimen [124]. Immunity has been shown to wane over time, and a booster dose is recommended 1–2 years after the primary dose to give long-term protective immunity [125].

Dengvaxia^®^ is a live-attenuated chimeric dengue vaccine that protects against disease from all four dengue virus (DENV) serotypes [95,126]. The genome of this live-attenuated vaccine is the 17D-204 backbone with the prM and E of YF replaced with those of the four wild-type DENV serotypes. The vaccine has been licensed as a three-dose regimen at zero, six, and 12 months in approximately 20 countries, but implementation has been slow due to the potential concerns of a live-attenuated dengue vaccine predisposing vaccinees to antibody-dependent enhancement (ADE). It was found that a small number of vaccinees who were infected with DENV after vaccination with Dengvaxia^®^, and who were naïve to DENV infection prior to vaccination, had a severe DENV infection resembling ADE. Due to this, Dengvaxia^®^ is only recommended for use in DENV-endemic areas in people between the ages of nine and 45 years who have evidence of previous DENV infection prior to vaccination. This is not straightforward, as flaviviruses are well known to serologically cross-react such that a positive DENV antibody titer may not be indicative of a previous DENV infection, but may represent a different flavivirus infection. Currently, the one of most concern is cross-reaction with Zika virus (ZIKV). Thus, development of diagnostics to support the use of Dengvaxia is focusing on a NS1-based diagnostic, because this protein has been shown to contain predominantly flavivirus-specific epitopes that do not cross-react between flaviviruses, including YF NS1, which is in the virus backbone of Dengvaxia.

Other flavivirus vaccine candidates are in development that use the ChimeriVax technology, including West Nile virus (WNV) and ZIKV. The first ChimeriVax WNV vaccine candidate, ChimeriVax-WN01, was composed of the 17D backbone with the prME of wild-type WNV strain WN NY99 [96,127]. ChimeriVax-WN01 was less neuroinvasive in mice than wild-type WNV, and less neurovirulent in mice and monkeys than 17D [96]. To further reduce the potential of neurovirulence in humans, mutations based on attenuating mutations in the live-attenuated JE vaccine strain SA14-14-2 were incorporated into the E protein gene of WNV (L107F, A316V, and K440R), thus generating ChimeriVax-WN02. The WNV vaccine candidate ChimeriVax-WN02 has advanced to phase II clinical trials, but to date has not advanced to phase III clinical trials. ChimeriVax-WN02 stimulated the production of neutralizing antibodies in the hamster and NHP models, and these animals were protected from challenge by WN NY99 [96,127,128]. Phase I and II clinical trials testing safety and immunogenicity showed no statistical difference in adverse events from ChimeriVax-WN02 when compared to placebo (NCT00746798 and NCT00442169) [97,98,129]. After a single dose of the vaccine candidate, most volunteers seroconverted by one month after vaccination, and all vaccinees were seropositive one year after vaccination [127]. Robust cytotoxic T-cell responses were also reported [130]. The sporadic nature of WNV outbreaks has made it difficult to obtain efficacy data on the path to licensure.

The ChimeriVax ZIKV vaccine candidate is known as ChimeriVax-Zika or CYZ [99]. Similar to the other ChimeriVax vaccines, this candidate is composed of the 17D backbone with the prME of ZIKV. In vitro, this chimeric virus showed evidence of attenuation, with slower replication kinetics in human neuronal cell culture than wild-type ZIKV. It was also less neurovirulent in mice than 17D. A129 mice inoculated with CYZ showed reduced viral loads in the organs, and no neuroinvasion was detected. The candidate vaccine induced high titers of neutralizing antibodies against ZIKV post-vaccination, and mice were subsequently protected from lethal challenge of ZIKV.

Another study, separate from the ChimeriVax platform, developed a chimeric ZIKV vaccine with replacement of 17D prME genes with those of ZIKV (YF-ZIKprM/E) that showed total protection against lethal challenge in mice with both ZIKV and YFV, suggesting the possibility that one vaccine could be used for both viruses [100]. They found that CD8^+^ T cells, not CD4^+^ T cells nor neutralizing antibodies, were what was required to protect against YFV with this candidate vaccine. It was also found that YF-ZIKprM/E could protect mouse fetuses from brain infections and malformations due to ZIKV [101].

As with all Zika vaccine candidates, clinical evaluation of chimeric 17D/ZIKV vaccine candidates has not been possible since the epidemic ended in 2017.

### 5.4. The 17D Vaccine as a Vector for Foreign Antigens as Vaccines

Due to its genetic stability, the 17D backbone has also been used to design vaccines for unrelated pathogens such as malaria, *Trypanasoma cruzi*, HIV, and Lassa virus. For malaria, a portion of the *Plasmodium yoelii* (closely related to *Plasmodium falciparum)* CS protein was inserted into the *fg* loop in EDII [102]. For *Trypanasoma cruzi*, the etiologic agent for Chagas disease, the amastigote surface protein-2 was inserted between the E and NS1 of 17DD [103]. The HIV vaccine candidate was generated by inserting SIVmac239 Gag sequences (known SIV T-cell epitopes) also between the E and NS1 of 17D [104]. Lastly, Lassa glycoproteins have been inserted into the C-terminal region of the 17D E protein to generate the chimeric Lassa vaccine candidate [105]. Many of these candidates are described in previously published articles [4].

Many of these vaccines and vaccine candidates work well because they stimulate both a humoral and a cellular immune response. Neutralizing antibodies are the recognized correlate of protection for the 17D vaccine, and it is unknown if a new vaccine construct would have the same correlate of protection qualitatively or quantitatively. If it did, would the neutralization titer associated with protective immunity be the same as 17D? However, it is important that new vaccine candidates for YF or any of the above diseases do not neglect cellular immunity, which is likely equally as important in developing an initial and memory response to YFV and/or the foreign antigen. Unfortunately, it is difficult to measure cellular immunity with a standardized, validated assay, as many labs use varying techniques to isolate and analyze T cells. Ideally, more emphasis will be put on understanding cell-mediated immunity after vaccination, as it is integral to both the cytotoxic response and an effective B-cell helper response, and may be critical for protective immunity induced by the foreign antigen.

## 6. Conclusions

The 17D vaccine has proved to be an excellent live-attenuated vaccine. However, there are problems of supply and demand because the vaccine is produced in embryonated chicken eggs. Any new vaccines against YFV should be able to be scaled up adequately, such that they can accommodate the global demand, which has recently increased due to a number of outbreaks in both Africa and South America, and will likely continue to increase with climate change and human encroachment into areas where the sylvatic cycle of YFV is present. Current WHO recommendations for the quality, safety, and efficacy of the yellow fever 17D vaccine require that only 17D live-attenuated vaccine can be used, grown only in embryonated chicken eggs, and evaluated for safety in NHPs only. These requirements would need to be reconsidered if new candidate vaccines are developed and require international approval. New vaccine platforms may be required to overcome additional or alternative regulatory hurdles, evaluation, and/or identification of a correlate of protection, and possibly new approaches of testing safety and efficacy in animal models.

One issue to overcome with the current live-attenuated 17D vaccines is the occurrence of serious adverse events (SAEs), including YEL-AVD and YEL-AND. These SAEs, while rare, are still concerns when it comes to YF vaccination [131].

There is no doubt that the use of embryonated chicken eggs to produce the vaccine is very dated; switching cell substrates has many potential advantages for manufacturing, but these may be tempered if the potency of the vaccine is reduced. This remains to be tested. In addition, there will be a need to undertake extensive safety evaluation of any vaccine produced in a different cell substrate. It is clear that an inactivated 17D vaccine would improve safety of the 17D vaccine, but it is likely that vaccinees would require more than one dose for it to be as effective as 17D. However, such inactivated vaccines may have a market in special populations who are unable to receive a live-attenuated vaccine.

If second-generation live-attenuated vaccines are explored, it is suggested that restricted genetic diversity would be an important criterion for such vaccine candidates. Restricted diversity lowers the likelihood of reversion to virulence, and is implicated in contributing to attenuation of many RNA viruses. Unfortunately, the molecular basis of attenuation of 17D is still poorly understood, which limits efforts to develop improved second-generation 17D vaccines or other live-attenuated vaccines produced using different platform technologies. Nonetheless, successes with reverse genetics, chimeric vaccines based on the 17D backbone, and PLLAVs inspire optimism that additional vaccines for infectious diseases based on the 17D vector will be developed in the future.

Most of the YF vaccine candidates discussed in this review have similar correlates of protection, which include high titers of neutralizing antibodies. Many of them prevent disease and mortality in animal models after virus challenge. Some of these candidates have moved on to non-inferiority trials in comparison to 17D. Even if a candidate vaccine is shown to be non-inferior to 17D, if it is to be implemented, it will still have to undergo discussions regarding cost-effectiveness, stability testing, global dissemination, national licensure, and likely WHO prequalification.

The WHO’s EYE initiative aims to eliminate YF epidemics by 2026, though the COVID-19 pandemic has negatively impacted the timeline for implementation of this goal. Nonetheless, the effects of COVID-19 are not all negative. The pandemic has shown the world that vaccines can be developed rapidly if sufficient funding is put toward the problem. New vaccine platforms have come to the forefront with development of COVID vaccines, and it is possible that one or more of these platforms could be used for a new-generation YF vaccine.

Until new YF vaccine candidates can be proven to be equally (non-inferior) or more effective (superior) at preventing YF disease and more cost-effective than 17D, the historic live-attenuated 17D vaccine is here to stay. New manufacturing protocols for 17D, new vaccine platforms for YFV, and chimeric vaccines using the 17D backbone are all important areas of research at the present time. The 17D vaccine is one of great historical significance, and it continues to have a large impact on vaccine research and public health today, a trend we believe will continue in the foreseeable future.

## Figures and Tables

**Table 1:** Amino acid substitutions and nucleotide changes between Asibi and 17D YF strains [4].

Structural Proteins		
*Nucleotide*	*Gene*	*AA Number (Asibi to 17D)*
854	M	L36F
1127	E	G52R
1482	E	A170V
1491	E	T173I
1572	E	K200T
1870	E	M299I
1887	E	S305F
2112	E	T380R
2193	E	A407V
**Nonstructural proteins**		
3371	NS1	I307V
3860	NS2A	M118V
4007	NS2A	T167A
4022	NS2A	T172A
4056	NS2A	S183F
4505	NS2B	I109L
6023	NS3	D485N
6876	NS4A	V146A
7171	NS4B	I95M
10,142	NS5	E836K
10,338	NS5	P900L
**3′ Untranslated Region Nucleotide changes (Asibi to 17D)**		
10,367		U → C
10,418		U → C
10,800		G → A
10,847		A → C

**Table 2:** Yellow fever vaccine candidates in development.

Vaccine Name [Reference]	Vaccine Type	Formulation/Makeup	Stage of Development	Cohort	Endpoints	Comments
XRX-001 [89]	Inactivated	YF-VAX inactivated with BPL adsorbed to aluminum hydroxide	Clinical: Phase I	60 healthy male and female volunteers	Safety: SAE incidence Efficacy: neutralizing antibody response	
VINFLAPI001/2010 [77]	Inactivated	Bio-Manguinhos/FIOCRUZ 17DD inactivated with BPL adsorbed to aluminum hydroxide	Pre-clinical	C57Bl/6 mice	Seroconversion of neutralizing antibodies and protection from lethal challenge	17DD grown in serum-free Vero cells in bioreactor
Chumakov Institute inactivated YF vaccine [78]	Inactivated	Chumakov Institute 17D-213 inactivated with BPL	Pre-clinical	BALB/c mice	Non-inferior immunogenicity compared to 17D	17D strain adapted to Vero cell culture
Recombinant vaccinia virus/17D YFV [90]	Replicating viral vector	YFV-specific cDNA clone 10III NS1-NS2A-NS2B expressed in wild-type vaccinia virus	Pre-clinical	BALB/c mice	Protection from lethal challenge	Only conferred partial immunity
MVA-YF and dVV-YF [80]	Non-replicating viral vector	Stamaril 17D prME expressed in modified vaccinia virus Ankara and D4R defective vaccinia virus	Pre-clinical	BALB/c mice	Safety, immunogenicity, and protection from lethal challenge	
MVA-BN-YF [75,91]	Non-replicating viral vector	17D prME expressed in modified vaccinia virus Ankara, Montanide ISA-720 adjuvant	Clinical: Phase I	Healthy adults aged 18–45 (NCT 02743455)	Safety, reactogenicity, immunogenicity	
pYF17D-16 iDNA [82]	DNA	PLLAV: iDNA plasmid containing 17D genome downstream of CMV promoter	Pre-clinical	AG129 and BALB/c mice	Safety and seroconversion of neutralizing antibodies, respectively	
pBeloBAC-FLYF and pBeloBAC-YF/ΔC [83]	DNA	PLLAV: plasmid containing 17D genome downstream of CMV promoter and upstream of hepatitis delta virus ribozyme and RNA pol II transcription terminator (ΔC: capsid gene deleted)	Pre-clinical	A129 mice	Seroconversion of neutralizing antibodies	
pShuttle/YFV-17D [84]	DNA	PLLAV: 17D-204 cDNA downstream of SV40 promoter, upstream of hepatitis delta virus ribozyme	Pre-clinical	AG129 mice	Measuring genetic diversity (safety correlate)	
p/YFE and pL/YFE [81]	DNA	DNA encoding 17DD E and E fused to LAMP-1, respectively (not PLLAV)	Pre-clinical	C57Bl/6 and BALB/c mice	Stimulation of T-cell responses, neutralizing antibodies; comparison to 17DD vaccination; protection from lethal challenge	
CJaYZ [85]	VLP	Tetravalent VLP against YFV, ZIKV, CHIKV, and JEV: CprME of the flaviviruses and C-E3-E2-E1 of CHIKV expressed on a lentiviral vector that has been stably expressed in 293 T cells under antibiotic selective pressure	Pre-clinical	BALB/c mice	Seroconversion of neutralizing antibodies	
(YF) prME mRNA [86]	RNA	YFV prME mRNA complexed with lipid derivatives	Pre-clinical	Cynomolgus macaques	Visualize vaccine trafficking dynamics to draining lymph nodes	No immunogenicity or efficacy data
Re-encoded wild-type YF viruses [87]	Synonymous transition mutations live-attenuated vaccine	Asibi and Ap7M (hamster-adapted) strains mutated to have 100–400 synonymous mutations in the NS2A-to-NS4B coding region of the YFV genome	Pre-clinical	Syrian golden hamsters (*M. auratus)*	Comparison of virulence and immunogenicity to wild-type/hamster-adapted YFV; protection from challenge	
YFE and YFE-LicKM [88]	Plant-produced subunit vaccine	E protein and E protein fused to bacterial enzyme lichenase produced by *Nicotiana benthamiana*	Pre-clinical	BALB/c mice	Seroconversion of neutralizing antibodies and protection from lethal challenge	
vYF-247 [70]	New manufacturing protocols	Stamaril and YF-VAX 17D genomes transfected into serum-free Vero cells; resulting seed lots grown in serum-free Vero cells	Pre-clinical	A129 and OF1 mice and Syrian golden hamsters (*M. auratus)*	Comparison to chicken embryo live-attenuated 17D in neurovirulence, viscerotropism, immunogenicity, protection from lethal challenge	
YFCEF-01-07 [92]	New manufacturing protocols	17DD grown in chicken embryo fibroblast culture	Pre-clinical	Swiss Webster mice and rhesus macaques	Immunogenicity and neurovirulence, respectively	

**Table 3:** Vaccine candidates in development that utilize yellow fever vaccine technology.

Vaccine Name [Reference]	Pathogen	Vaccine Formulation	Stage of Development	Cohort	Endpoints	Comments
Imojev™ (JE-CV) [93,94]	JEV	prME proteins of JEV SA14-14-2 in 17D backbone	Licensed	14 countries		Produced in Vero cells
Dengvaxia^®^ [95]	DENV1-4	17D-204 backbone with the prM and E of YF replaced with those of the four wild-type DENV serotypes	Licensed	20 countries		
ChimeriVax-WN01 [96]	WNV	17D backbone with WN NY99 prME	Pre-clinical	ICR mice and rhesus macaques	Reduced neurovirulence and neurotropism when compared to wild-type WNV	
ChimeriVax-WN02 [96,97,98]	WNV	Same as WN01 with added mutations in E: L107F, A316V, and K440R	Clinical: Phase II	Healthy adults aged 18–40 years (NCT00442169); adults over 50 years of age (NCT00746798)	Testing safety and immunogenicity (seroconversion of neutralizing antibodies) of low, medium, and high doses	
ChimeriVax-Zika (CYZ) [99]	ZIKV	17D backbone with prME of ZIKV	Pre-clinical	A129 mice	Reduced viral loads, reduced neurovirulence/neuroinvasion, seroconversion of neutralizing antibodies, protection from lethal challenge	
YF-ZIKprM/E [100,101]	ZIKV	17D backbone with prME of ZIKV	Pre-clinical	AG129, IFNAR1^−/−^, C57Bl/6, BALB/c, and immunocompetent NMRI mice	Protection from lethal challenge; protection from brain infections and malformations in mouse fetuses	
17D/13 and 17D/8 [102]	*Plasmodium falciparum*	SYVPSAEQI portion of *Plasmodium yoelii* CS protein inserted into the *fg* loop in EDII	Pre-clinical	Rhesus macaques	Monkey neurovirulence test	
YF17D/ENS1/Tc [103]	*Trypanasoma cruzi*	Amastigote surface protein-2 inserted between E and NS1 of 17DD	Pre-clinical	A/J mice	Seroconversion of neutralizing antibodies	
rYF17D/SIVGag_45–269_ [104]	HIV	SIVmac239 Gag sequences inserted between E and NS1 of 17D	Pre-clinical	Rhesus macaques	Generation of CD8^+^ T-cell responses	
YFV17D/LASV-GPC [105]	Lassa virus	Lassa glycoproteins inserted into the C-terminal region of the 17D E protein	Pre-clinical	Strain 13 guinea pigs	Seroconversion of antibodies; protection from lethal challenge	
YF-S0 [106]	SARS-CoV-2	Non-cleavable prefusion spike protein of SARS-CoV-2 inserted between E and NS1 or 17D	Pre-clinical	Syrian golden hamsters (*M. auratus),* AG129 hamsters, STAT2^−/−^ hamsters; BALB/c and IFNAR1^−/−^ mice, cynomolgus macaques	Safety, immunogenicity (neutralizing antibodies), efficacy; protection from infection/lung disease with SARS-CoV-2	

## Data Availability

Not applicable.
